# Metachronous Brain Metastasis in patients with EGFR-mutant NSCLC indicates a worse prognosis

**DOI:** 10.7150/jca.46462

**Published:** 2020-10-21

**Authors:** Wen Ouyang, Jing Yu, Yan zhou, Yu Xu, Jie Li, Jun Gong, Junhong Zhang, Conghua Xie

**Affiliations:** 1 Department of Radiation and Medical Oncology, Zhongnan Hospital, Wuhan University, Wuhan, China.; 2 Hubei Key Laboratory of Tumor Biological Behaviors, Zhongnan Hospital, Wuhan University, Wuhan, China.; 3 Hubei Clinical Cancer Study Center, Zhongnan Hospital of Wuhan University, Wuhan, China.

**Keywords:** non-small cell lung cancer, epidermal growth factor receptor, brain metastases, synchronous, metachronous

## Abstract

**Purpose:** NSCLC patients with EGFR mutation were associated with high incidence of brain metastasis (BM). BM could be grouped by the time of occurrence, including synchronous BM at initial diagnosis and metachronous BM during disease course. The primary aim of the study was to investigate the survival of patients with metachronous BM.

**Methods:** A total of 99 EGFR-mutant advanced NSCLC patients in our institute between 2012 and 2018 were grouped into synchronous BM and metachronous BM. Comparisons of OS were performed based on BM status. The independent prognostic factors of OS were investigated, and extracranial and intracranial PFS were further analyzed.

**Results:** Patients with metachronous BM (mOS: 22.1 months) had poorer outcomes than synchronous BM (mOS: 30.3 months) (*P*=0.016). Moreover, multivariate analysis indicated that BM status (*P*=0.015), local therapy for BM (*P*=0.013) and subsequent treatment of Osimertinib (*P*=0.008) impact significantly on OS. Significantly, the proportion of local therapy for BM had no difference between patients with synchronous and metachronous BM. And patients with metachronous BM harbored a more favorable prognostic factor (higher proportion of subsequent Osimertinib treatment), but also harbored a poorer prognostic factor (metachronous BM), which confirmed BM status was the most significant prognostic factor of OS. At last, results of extracranial and intracranial PFS indicated that patients with metachronous BM tended to have a higher risk of intracranial disease progression.

**Conclusions:** Patients developing metachronous BM during EGFR-TKIs treatment have worse outcomes than synchronous BM. Our findings suggested that the patients with metachronous BM should receive more aggressive treatments.

## Introduction

Non-small cell lung cancer (NSCLC) is one of the malignancies with the highest morbidity and mortality 1. Brain is a frequent site of NSCLC metastasis, and patients with brain metastasis (BM) are associated with high mortality and poor life quality 2. The median survival in patients who suffer BM without treatment is less than 1 month, and the median survival after whole brain radiation therapy (WBRT) is only 3-6 months 3. Additionally, BM could be grouped by the time of occurrence, including synchronous BM and metachronous BM. Approximately 10% of NSCLC patients are synchronous BM at their initial diagnosis, and 40-50% of patients develop metachronous BM during the course of the disease 4.

Epidermal growth factor receptor (EGFR) mutation is a favorable prognostic marker of NSCLC. EGFR-tyrosine kinase inhibitors (TKIs) largely improved the survival of EGFR-mutant advanced NSCLC patients 5-7. However, patients with EGFR mutation were associated with higher incidence of developing BM compared to patients with wild type EGFR 8-10. Moreover, the longer survival of patients with EGFR mutant NSCLC is associated with higher exposure risk to BM 8. Therefore, patients with EGFR mutant NSCLC tend to have a high risk of developing metachronous BM. Phase III trials of EGFR-TKIs for EGFR-mutant advanced NSCLC reported patients with BM had a worse median overall survival (OS) than the patients without BM, but synchronous BM and metachronous BM are seldom differentiated in these trials 11-13. There is a lack of sufficient data on the prognosis of metachronous BM among EGFR-mutant advanced NSCLC patients. Therefore, it was necessary to evaluate the prognosis of EGFR-mutant advanced NSCLC patients with metachronous BM.

Consequently, we established a retrospective single-institutional database including consecutive EGFR-mutant advanced NSCLC patients with synchronous BM or metachronous BM from January 2012 and June 2018, to evaluate the survival of patients grouped by BM status, to explore the prognostic factors of OS, and to analyze potential mechanism of different outcomes based on BM status.

## Methods

### Patients

Between January 2012 and June 2018, a total of 229 consecutive patients with EGFR-mutant advanced NSCLC were included at the Department of Radiation and Medical Oncology, Zhongnan Hospital of Wuhan University. Among them, three patients were excluded due to short EGFR-TKI treatment (< 1 month), and 127 patients without BM were excluded. A total of 99 eligible patients with synchronous or metachronous BM were enrolled in this study. Our inclusion criteria are: (1) NSCLC was confirmed by cytology (8 pts), or histology (91 pts) (World Health Organization, WHO); (2) EGFR mutations were confirmed by real-time quantitative PCR (ARMS, 83 pts) or Next Generation Sequencing (NGS, 16 pts), using histological or cytological specimens from primary or metastatic lesions; (3) The disease was clinically diagnosed as stage IIIB (2 pts)-IV (97 pts) (American Joint Committee on Cancer, the 7th Edition); (4) The patients were treatment naive for EGFR-TKIs treatment; (5) All patients received brain Magnetic Resonance Imaging (MRI) scans within 1 month before EGFR-TKIs treatment. A total of 69 patients were synchronous BM at initial diagnosis, and 30 patients developed metachronous BM during EGFR-TKIs treatment. The clinical and therapeutic characteristics of these included patients grouped by BM status are shown in **Table 1.**

### Treatment and follow up

Among the 99 patients with synchronous or metachronous BM, 16 patients received chemotherapy as their first-line therapy, and the other 83 patients received EGFR-TKIs treatment initially. EGFR-TKIs (gefitinib, erlotinib, or icotinib) were continuously administered until progression of disease (PD) or intolerable side effects. Treatment beyond PD was allowed on the judgement of continuously clinical benefit by the oncologists.

Follow-up examinations were performed every 2 months, including thoracic and abdominal CT scans, brain MRI scans. Progression-free survival (PFS) was defined as the time from EGFR-TKIs treatment to PD (including local, regional, or distant progression) or death from any cause. OS was defined as the time from EGFR-TKIs treatment to death from any cause. Extracranial PFS was defined as the time from EGFR-TKIs treatment to extracranial PD (excluding intracranial PD) or death from any cause. Intracranial PFS (iPFS) was defined as the time from EGFR-TKIs treatment to intracranial PD (including appearance of new intracranial lesions, or existing intracranial lesions progression) or death from any cause. Intracranial PFS after brain radiotherapy (BRT) was defined as the time from radiotherapy for intracranial lesions (71 patients who received BRT) or from EGFR-TKIs treatment (28 patients who didn't receive BRT) to intracranial PD or death from any cause. Treatment responses were evaluated by the response evaluation criteria in solid tumors as complete response (CR), partial response (PR), stable (SD), and progression (PD).

### Statistics

All statistical analyses were conducted using Statistical Package for Social Scientists (SPSS/Windows, Version 22.0, SPSS Inc., Chicago, USA). Descriptive statistics were used for categorical variables (frequency and percentage) and continuous variables (median and range). The cumulative incidence of survival was calculated by the Kaplan-Meier method with 95% confidence intervals (CIs). Univariable and multivariable Cox regression analyses were performed to explore the prognostic factors associated with OS. The multivariable Cox regression analysis simultaneously included those factors that had shown associations (*P* < 0.100) in the univariable Cox regression analyses. All tests were two-sided and *P* < 0.05 were considered statistically significant.

## Results

### Patient characteristics

A total of 99 eligible patients with BM were analyzed in the retrospective study. All patients received brain MRI scans before EGFR-TKIs treatment. Among them, 69 patients were confirmed as synchronous BM at initial diagnosis, the other 30 patients showed negative results of brain MRI scans before EGFR-TKIs treatment, whereas developed metachronous BM during EGFR-TKIs treatment. The clinical and therapeutic characteristics of these patients grouped by BM status are shown in **Table 1**. The median age of the patients that would develop metachronous BM and patients with synchronous BM were both 54 years. There was no difference between the two groups with respect to gender, histology, BMI, smoking status, tumor markers levels, clinical stages, extracranial metastases, type of EGFR mutations, type of EGFR-TKIs, diagnosed as oligometastasis or not, and whether local therapy for BM or primary lesion. However, due to our study is a limited-sample retrospective study, the distribution of KPS score and subsequent treatment of Osimertinib between the two groups was imbalanced (KPS score ≥ 80: 100% patients that would develop metachronous BM vs. 84.1% patients with synchronous BM; and subsequent treatment of Osimertinib: 43.3% patients that would develop metachronous BM vs. 23.2% patients with synchronous BM). Additionally, the proportion of symptomatic BM in patients with metachronous BM (30%) was lower than synchronous BM (49.3%).

### Overall survival of patients grouped by BM status

For 99 EGFR-mutant advanced NSCLC patients with BM, the median duration of follow-up was 22.8 months (95% CI: 17.5-28.1 months). The median OS of these patients was 29.3 months (95% CI: 19.5-39.1 months). The 1-, 2- and 3-year OS rates were 91.5%, 55.4% and 33.4% respectively.

**Figure 1:**
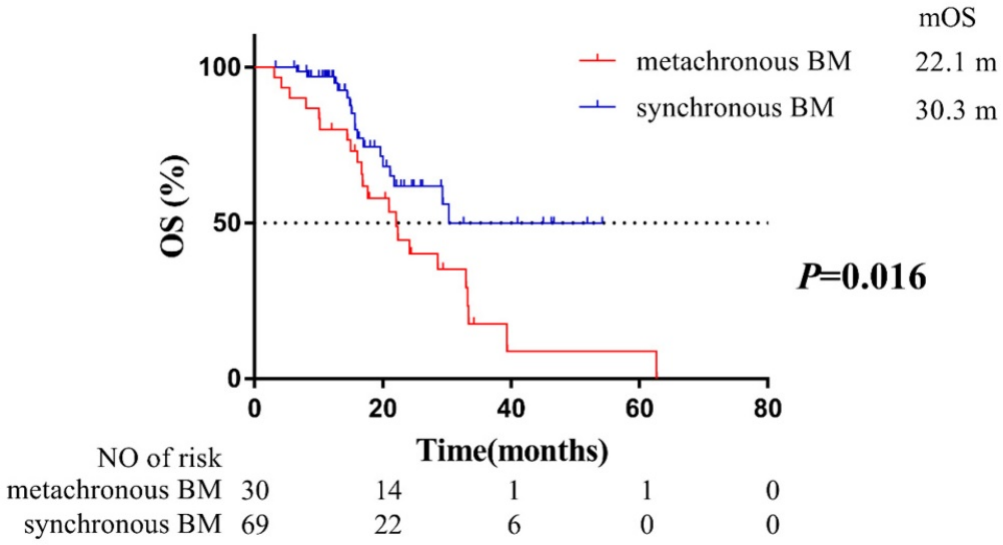
Kaplan-Meier plot of OS in patients with EGFR-mutant advanced NSCLC grouped by BM status. OS, overall survival; NSCLC, non-small cell lung cancer; BM, brain-metastases; CI, confidence interval.

To evaluate the impact of BM status on OS, these 99 patients were grouped by synchronous BM and metachronous BM. Compared with patients with synchronous BM, patients developing metachronous BM during the course of EGFR-TKIs treatment were at a higher risk on OS (HR=2.17, 95% CI: 1.14-4.12; median OS: 22.1 months of patients with metachronous BM vs. 30.3 months of patients with synchronous BM,** Figure 1**). Our findings indicated that metachronous BM was a significantly poor prognostic factor for OS.

### Univariate and Multivariate analysis of OS

Then 99 patients were further analyzed to explore the prognostic factors associated with OS by univariable and multivariable Cox regression analyses, which were shown in **Table 2**. In univariate Cox regression analyses, BM status, bone metastasis, local therapy for BM, subsequent treatment of Osimertinib significantly influenced OS (P < 0.05) (**Table 2**). Other factors such as gender, KPS score, tumor markers levels before treatment, the first-line treatment regimen, type of EGFR mutations, number of metastases, and diagnosed as oligometastasis were not found to be statistically significant (P > 0.1) (**Table 2**).

Multivariable Cox regression analysis simultaneously included those factors that had shown associations (*P* < 0.100) in the univariable Cox regression analyses. Moreover, results of multivariable Cox regression analyses showed that BM status (*P* =0.015), local therapy for BM (*P* =0.013) and subsequent treatment of Osimertinib (*P* =0.008) had significant impact on OS (**Table 2**).

### Progression-free survival of patients grouped by BM status

**Figure 2:**
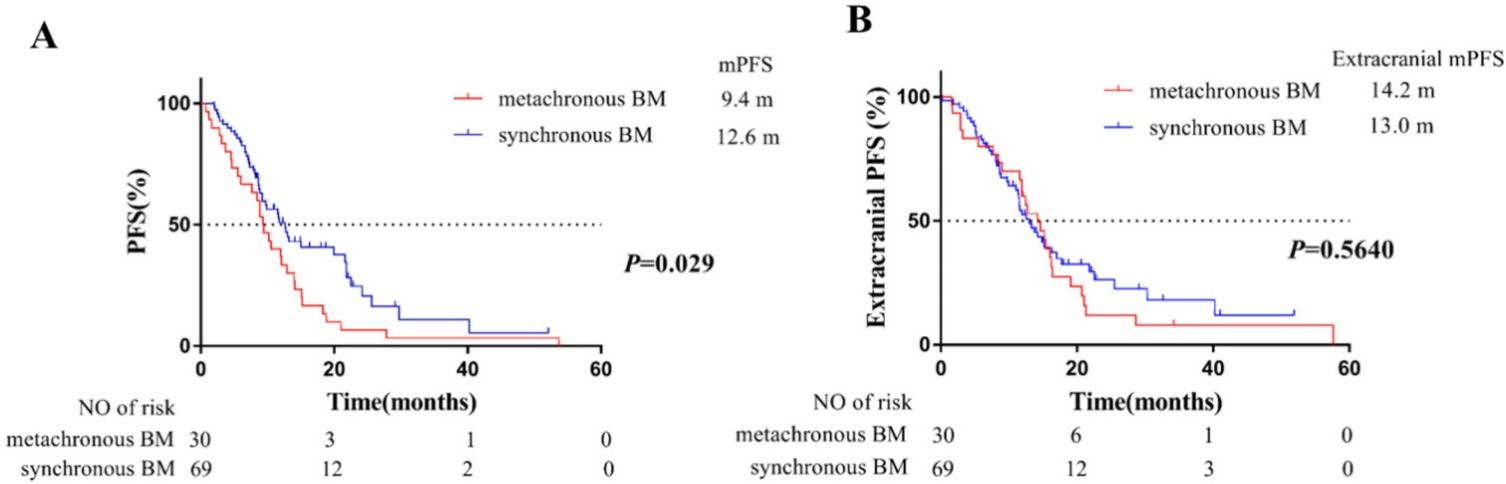
Kaplan-Meier plot of PFS (**A**) and extracranial PFS (**B**) in EGFR-mutant advanced NSCLC patients grouped by BM status. PFS, progression-free survival, the time from the EGFR-TKI treatment to PD or death; extracranial PFS, the time from EGFR-TKIs treatment to extracranial PD (exclude intracranial PD) or death from any cause; NSCLC, non-small cell lung cancer; BM, brain-metastases; CI, confidence interval.

For PFS, a total of 76 patients progressed during follow-up time, whereas 40 patients first progressed in intracranial disease. Among them, this consisted of 20 patients with synchronous BM (20/69, 29.0%), and 20 of patients with metachronous BM (20/30, 66.7%). The median PFS of these 99 patients was 11.5 months (95% CI: 8.7-14.2 months). Compared with patients with synchronous BM, patients developing metachronous BM during the course of EGFR-TKIs treatment were at higher risk on PFS (HR=1.67, 95%CI:1.05-2.66). In detail, the median PFS of 69 patients with synchronous BM and 30 patients with metachronous BM was 12.6 months and 9.4 months, respectively (**Figure 2A**).

For extracranial PFS, 73 patients progressed during follow-up time. The median extracranial PFS of 69 patients with synchronous BM and 30 patients with metachronous BM was 13.0 months and 14.2 months, respectively. However, there was no significant difference between the two groups (*P* =0.56) (**Figure 2B**).

### Intracranial progression-free survival of patients grouped by BM status

**Figure 3:**
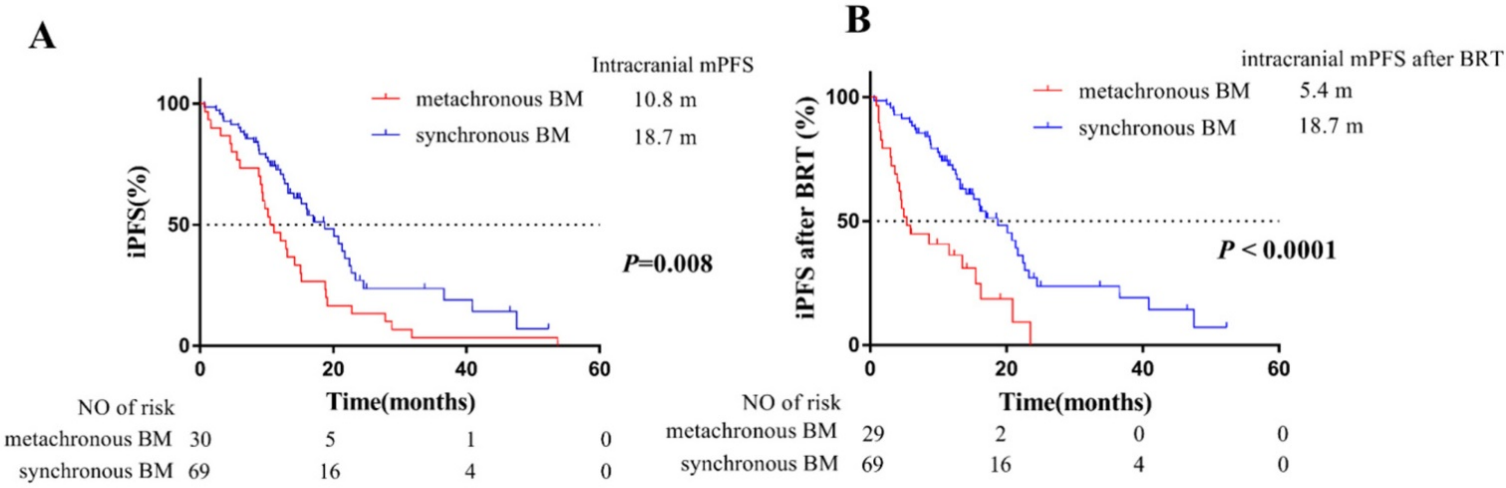
Kaplan-Meier plot of iPFS (**A**) and iPFS after BRT (**B**) in EGFR-mutant advanced NSCLC patients grouped by BM status. iPFS, intracranial PFS, the time from EGFR-TKIs treatment to intracranial PD (including appearance of new lesions, or existing lesions progression) or death from any cause; BRT, brain radiotherapy; iPFS after BRT, the time from radiotherapy for intracranial lesions (71 patients who received BRT) or from EGFR-TKIs treatment (28 patients who didn't receive BRT) to intracranial PD or death from any cause; NSCLC, non-small cell lung cancer; BM, brain-metastases; CI, confidence interval.

For intracranial PFS, a total of 70 patients progressed during follow-up time. The median intracranial PFS of 69 patients with synchronous BM and 30 patients with metachronous BM was 18.7 months and 10.8 months, respectively. It was indicated that patients developing metachronous BM during the course of EGFR-TKIs treatment were at higher risk on iPFS than synchronous BM (*P* = 0.008, **Figure 3A**).

Additionally, there were 71 patients received brain radiotherapy (BRT). Among them, 30 patients received WBRT and 19 patients received stereotactic radiosurgery (SRS) of 69 patients with synchronous BM, and 14 patients received WBRT and 8 patients received SRS of 30 patients with metachronous BM (**Table 1**). The median intracranial PFS after BRT of 69 patients with synchronous BM and 30 patients with metachronous BM was 18.7 months and 5.4 months, respectively. Our findings also confirmed that patients developing metachronous BM during EGFR-TKIs treatment had poorer responsiveness to BRT than patients with synchronous BM (*P* < 0.001, **Figure 3B**).

## Discussion and Conclusion

During the past two decades, the advances of EGFR-TKIs revolutionarily improved the prognosis of EGFR-mutant advanced NSCLC patients. The clinical trials of the first- or second-generation EGFR-TKIs showed a median OS of 19.3-33.2 months 14,15. The prognosis of patients with BM has been considered uniformly poorer than patients without BM 3. Our results of 99 EGFR-mutant advanced NSCLC patients with BM (including synchronous and metachronous) indicated a median OS of 29.3 months. The OS of patients with BM in our study is roughly similar to the overall patients set including non-BM subset in the first-generation EGFR-TKIs clinical trials 14. It might be attributed largely to the subsequent usage of third-generation EGFR-TKIs for patients with acquired T790M mutation (**Table 1**).

Although EGFR-TKIs was reported to be more effective for BM than chemotherapy in EGFR-mutant NSCLC patients 14, there remain some patients developing metachronous BM during the course of EGFR-TKIs therapy. Lee et al found that 26% of the patients developed central nervous system (CNS) failure among 166 patients with a clinical benefit to first-generation EGFR-TKIs (gefitinib or erlotinib) treatment 16. Moreover, compared with NSCLC patients with wild type EGFR, EGFR-mutant patients with longer survival exposed to BM 8, thus patients with EGFR mutation were associated with higher incidence of developing metachronous BM 17. Therefore, it was suggested more attention should be paid for metachronous BM of patients with EGFR mutant NSCLC whereas there is a lack of sufficient data. To evaluate the impact of BM status on OS, the 99 patients were grouped into synchronous BM and metachronous BM. Our findings first confirmed that patients developing metachronous BM during EGFR-TKIs treatment had poorer outcomes than patients with synchronous BM at initial diagnosis (*P* = 0.016, **Figure 1**).

However, it was also reported that clinical and therapeutic characteristics such as KPS score, local therapy for BM, radiotherapy for primary lesion 18, subsequent treatment of Osimertinib 19, and diagnosed as oligometastasis 20 could influence OS. To eliminate the impact of potential confounders, and to further confirm metachronous BM was an independent prognostic factor of poorer OS, we performed multivariable Cox regression analysis to investigate the potential prognostic factors. Results of multivariable Cox regression analysis indicated that BM status (*P* =0.015), local therapy for BM (*P* =0.013) and subsequent treatment of Osimertinib (*P* =0.008) significantly impacted on OS (**Table 2**). Significantly, the proportion of local therapy for BM (either WBRT or SRS) had no difference between patients with synchronous and metachronous BM (**Table 1**). Despite patients with metachronous BM harbored a more favorable prognostic factor (higher proportion of subsequent treatment of Osimertinib, **Table 1**), but also harbored a poorer prognostic factor, which was metachronous BM. As a result, our results ultimately confirmed that patients that would develop metachronous BM might harbor a more favorable prognostic factor but still indicated a poorer OS than patients with synchronous BM (**Figure 1**), which confirmed that BM status was the most significant prognostic factor of OS.

Furthermore, we evaluated the PFS of patients grouped by BM status. Our results showed that the median PFS of the overall 99 patients was 11.5 months, which was consistent with the PFS of the first-generation EGFR-TKIs clinical trials 21. Patients with metachronous BM were also at higher risk on PFS (HR=1.67, 95% CI: 1.05-2.66) (**Figure 2A**). However, for extracranial PFS, our results showed there was no significant difference between the two groups (*P* =0.56) (**Figure 2B**), which was suggested that the responsiveness of extracranial lesions to EGFR-TKIs was similar between the two groups. In addition, compared with synchronous BM, higher proportion of patients with metachronous BM (20/30, 66.7%) first progressed in intracranial disease. Therefore, our results confirmed that patients of metachronous BM group tended to have a higher risk of intracranial disease progression. It was known that the brain radiotherapy could improve blood-brain barrier (BBB) permeability of EGFR-TKIs. Therefore, the earlier intervene of radiotherapy for BM in synchronous BM group might partly explained worse outcomes in metachronous BM group.

Our results of median intracranial PFS also confirmed patients of metachronous BM group have a higher risk of intracranial disease progression. In detail, patients developing metachronous BM during EGFR-TKIs treatment had a shorter median iPFS of 10.8 months than patients with synchronous BM of 18.7 months (*P* = 0.008, **Figure 3A**). Then the median iPFS after BRT of 69 patients with synchronous BM and 30 patients with metachronous BM is 18.7 months and 5.4 months, respectively (*P* < 0.001, **Figure 3B**). It was further confirmed that metachronous BM occurred during EGFR-TKIs treatment had a worse responsiveness to BRT, compared with synchronous BM. The poorer iPFS after BRT partly resulted in a poorer OS of patients with metachronous BM. Therefore, for EGFR-mutant advanced NSCLC, our findings suggested that the patients without BM at initial diagnosis but would develop metachronous BM during the course of treatment harbored a worse intracranial responsiveness to EGFR-TKIs, and those patients should receive more aggressive treatments. And we could recommend patients with high risk of developing metachronous BM to receive the first-line Osimertinib treatment 13 or prophylactic cranial irradiation (PCI) 22.

In conclusion, the findings of this study are as follows. First, our studies firstly confirmed EGFR-mutant advanced NSCLC patients with metachronous BM had worse outcomes than synchronous BM. Second, the multivariate Cox analysis confirmed that BM status was an independent prognostic factor of OS. Third, our results of extracranial and intracranial PFS confirmed that patients of metachronous BM group tended to have a higher risk of intracranial disease progression. Consequently, our findings suggested that the patients without BM at initial diagnosis but developing metachronous BM during the course of EGFR-TKIs treatment should receive more aggressive treatments. Certainly, there are several limitations in our study that included a retrospective study in a single institution, which inevitably resulted in a selection bias. More finely devised prospective and random study is needed to validate the conclusion.

## Figures and Tables

**Table 1:** Baseline and treatment characteristics of patients grouped by BM status

Characteristic	Patients developing metachronous BM (n=30)		Patients with synchronous BM (n=69)	
	NO.	%	NO.	%
**Age, years**				
Median (Range)	54 (33-75)		54 (36-73)	
**Gender**				
Male	14	46.7	22	31.9
Female	16	53.3	47	68.1
**KPS score**				
≥80	30	100	58	84.1
<80	0	0	11	15.9
**Histology**				
Adenocarcinoma	28	93.3	66	95.7
Non-adenocarcinoma	2	6.7	3	4.3
**BMI**				
Mean (95%CI)	22.7 (16.3-29.2)		22.0 (16.3-27.7)	
**Smoking status**				
Yes	8	26.7	12	17.4
No	22	73.3	57	82.6
**CEA (ng/ml)**				
Median (Range)	30.5 (1.5-1819)		21.5 (0.6-2278)	
CA125 (ng/ml)				
Median (Range)	69.4 (11.3-954.5)		37.0 (5.5-5304)	
NSE (ng/ml)				
Median (Range)	15.2 (7.6-55.2)		13.8 (6.7-70.9)	
**First-line treatment regimen**				
EGFR-TKI treatment	21	70	62	89.9
Chemotherapy	9	30	7	10.1
**Type of EGFR mutations**				
Exon 21 point	14	46.7	25	36.2
Exon 19 deletion	11	36.7	36	52.2
Other	5	16.7	8	11.6
**NO. of extracranial metastases**				
0	2	6.7	0	0.0
1	14	46.7	26	37.7
2	10	33.3	23	33.3
3 or more	4	13.3	20	29.0
**Clinical stages**				
Stage IIIB	2	6.7	0	0.0
Stage IV	28	93.3	69	100
**Location of extracranial metastatic sites**				
Pleural effusion	6	20.0	2	2.9
Liver	4	13.3	6	8.7
Adrenal	1	3.3	9	13.0
Bone	18	60	27	39.1
Lung	17	56.6	25	36.2
Other	2	6.7	7	10.1
**Type of EGFR-TKIs**				
Gefitinib	19	63.3	44	63.8
Erlotinib	7	23.3	19	27.5
Icotinib	4	13.4	6	8.7
Symptomatic BM	9	30.0	34	49.3
**Local therapy for BM**				
None	8	26.7	20	29.0
WBRT	14	46.7	30	43.5
SRS	8	26.6	19	27.5
Radiotherapy for primary lesion	10	33.3	19	27.5
Subsequent treatment of Osimertinib	13	43.3	16	23.2
Diagnosed as oligometastasis	7	23.3	17	24.6

Abbreviations: BM, brain metastasis; KPS, Karnofsky performance status; BMI, body mass index; CEA, carcino-embryonic antigen; CA125, carbohydrate antigen 125; NSE, neuron-specific enolase; EGFR, epidermal growth factor receptor; TKI, tyrosine kinase inhibitor; WBRT, whole brain radiation therapy; SRS, stereotactic radiosurgery.

**Table 2:** Univariate and multivariate analyses for the factors associated with OS

Factors	Univariate analysis of OS (%)			Multivariate analysis of OS (%)		
	HR	95% CI	*P*	HR	95% CI	*P*
**BM status**						
Synchronous *VS* metachronous	0.462	0.243-0.878	0.018	0.426	0.214-0.846	0.015
Gender: female *VS* male	0.801	0.420-1.526	0.500			
Age, years	0.972	0.942-1.003	0.081	0.971	0.941-1.003	0.071
KPS score: ≥80 *VS* <80	0.728	0.221-2.390	0.600			
BMI	1.010	0.901-1.132	0.865			
Smoking: yes *VS* no	0.758	0.323-1.722	0.508			
**Tumor markers level before treatment**						
CEA (ng/ml)	1.000	0.999-1.001	0.601			
CA125 (ng/ml)	1.000	0.999-1.001	0.775			
NSE (ng/ml)	1.014	0.988-1.041	0.297			
**First-line treatment regimen**						
Chemotherapy *VS* EGFR-TKIs	1.389	0.672-2.869	0.375			
**Type of EGFR mutations**			0.440			
19-del *VS* L858R	1.545	0.748-3.194	0.240			
Other *VS* L858R	1.724	0.586-5.073	0.323			
**Type of EGFR-TKIs**			0.268			
Erlotinib *VS* Gefitinib	0.614	0.215-1.753	0.362			
Icotinib *VS* Gefitinib	0.456	0.158-1.317	0.147			
**NO. of extracranial metastasis**						
0-2 *VS* 3 or more	0.523	0.181-1.514	0.232			
**Location of extracranial metastasis**						
Pleural effusion	1.655	0.870-3.148	0.125			
Liver	0.779	0.186-3.255	0.732			
Adrenal	1.097	0.337-3.578	0.877			
Bone	2.027	1.062-3.868	0.032	1.659	0.822-3.350	0.158
Retroperitoneal lymph node	2.305	0.888-5.984	0.086	1.354	0.493-3.720	0.556
Other	1.793	0.544-5.916	0.337			
Symptomatic BM yes *VS* no	0.523	0.259-1.057	0.071	1.294	0.537-3.116	0.565
Local therapy for BM	0.478	0.244-0.935	0.031	0.357	0.159-0.805	0.013
Radiotherapy for primary lesion	1.741	0.906-3.345	0.096	1.437	0.665-3.105	0.356
Subsequent treatment of Osimertinib	0.442	0.213-0.918	0.029	0.337	0.151-0.751	0.008
Diagnosed as oligometastasis	0.531	0.222-1.273	0.156			

Abbreviations: OS, overall survival; HR, hazard ratio; CI, confidence interval; BM, brain metastasis; KPS, Karnofsky performance status; BMI, body mass index; CEA, carcino-embryonic antigen; CA125, carbohydrate antigen 125; NSE, neuron-specific enolase; EGFR, epidermal growth factor receptor; TKI, tyrosine kinase inhibitor.
